# An Exponential Curve Relationship Between Serum Urate and Migraine: A Cross-Section Study From NHANES

**DOI:** 10.3389/fneur.2022.871783

**Published:** 2022-04-15

**Authors:** Peiwei Hong, Yao Liu, Yang Wan, Hai Xiong, Yanming Xu

**Affiliations:** ^1^Department of Neurology, West China Hospital, Sichuan University, Chengdu, China; ^2^Department of Geriatric Medicine and Neurology, West China School of Public Health and West China Fourth Hospital, Sichuan University, Chengdu, China; ^3^Xindu Hospital of Traditional Chinese Medicine, Chengdu Medical College, Chengdu, China; ^4^Medical College of Tibet University, Lhasa, China

**Keywords:** migraine, headache, serum urate, NHANES, cross-section study

## Abstract

**Background:**

Migraine is a common neurological disease and an important cause of disability worldwide. Serum urate is the end product of purine metabolism in Homo sapiens and other hominoids. Previous studies about the serum urate level in migraine were contradictory. Hence, we present a cross-section study to clarify the association between serum urate and migraine and explore the dose effect of serum urate on migraine.

**Materials and Methods:**

The data for this cross-section study were acquired from the National Health and Nutrition Examination Survey (NHANES). A diagnosis of migraine was made through patient the self-reported and prescription medication. For data analysis, the weighted linear regression model, weighted chi-square test, logistic regression models, smooth curve fittings, and the two-piecewise linear regression model were utilized for data analysis. All data analysis was conducted on Empower software.

**Results:**

Totally, 18,637 participants were enrolled in this study, of which 208 were migraineurs. The rest were set as control. There existed a statistically significant difference in mean age (*p* = *0.0389*), gender (*p*< *0.0001*), race (*p*< *0.0001)*, data release cycle (*p* = *0.048*), drug usage, blood albumin (*p*< *0.0001*), blood total protein (*p*< *0.0001*), hemoglobin (*p*< *0.0001*), serum iron (*p*< *0.0001*), and serum urate (*p*< *0.0001*) between the two groups. According to logistic regression models, there existed no consistent linear relationship between serum urate and migraine before (model 1: odd ratio (*OR*) = 0.83, *p* = *0.0004*) or after adjusting for confounders (model 2: *OR* = 0.96, *p* = 0.5198; model 3: *OR* = 0.84, *p* = 0.0184). However, smooth curve fittings found an exponential curve relationship between serum urate and migraine. Furthermore, when serum urate was more than 7.8 mg/dl, higher serum urate was correlated with higher migraine occurrence (model 1: *OR* = 1.54, *p* = 0.0022; model 2: *OR* = 1.51, *p* = 0.0050; model 3: *OR* = 1.77, *p* = 0.0348). Besides, 8 out of the 208 migraineurs had a serum urate higher than 7.8 mg/dl.

**Conclusions:**

In conclusion, there existed an exponential curve relationship between serum urate and migraine, with an infliction point of 7.8 mg/dl. When serum urate was more than 7.8 mg/dl, increased serum urate was correlated with higher migraine occurrence.

## Introduction

Migraine is a common neurological disease and an important cause of disability worldwide, whose years of life lived with disability is 45.1 million and disability-adjusted life-years is 1.9% (1, 2). It is characterized by a recurrent, unilateral, moderate or severe, pulsating headache. The headache attack may last 4–72 h and be associated with nausea and/or phonophobia/photophobia (2).

Currently, migraine is considered an energy deficit syndrome partially due to mitochondrial dysfunctions (3). Besides, it is a complex neuroinflammatory disorder involving predominant activation of the trigeminovascular system with unclear molecular mechanisms (3, 4). These metabolic factors include behavioral factors, environmental factors, dietary triggers, hormonal changes, and genetic changes (3, 5). Oxidative stress is the imbalance between oxidation and antioxidation, which might be influenced by metabolic factors. Some migraineurs showed lower activity of catalase, non-oxidized thiol concentration, and total antioxidant capacity in serum (6, 7). Meanwhile, migraineurs showed the decreased activity of superoxide dismutase in their platelets and erythrocytes (7, 8).

Serum urate is the end product of exogenous and endogenous purine metabolism in Homo sapiens and other hominoids, which acts as an antioxidant *in vivo* and is associated with oxidative stress (9). It had been found that serum urate could lead to an increase in oxidative stress levels in a manner independent of xanthine oxidoreductase activity, especially in female (9). The gender-specific relationship of serum urate with oxidative stress might be due to the difference of serum estrogen level (10). Furthermore, serum urate showed lower levels and was posited as a neuroprotective agent in some neurological disorder (9, 11–13). On the contrary, some studies found that serum urate might act as a pro-oxidant, which might promote the oxidation stress (13). In addition, the contradiction of anti-oxidant effects and pro-oxidant effects might be due to the dose effect of serum urate (13). A previous study showed that a lower serum urate level was found in migraine, which was not influenced by the subtypes of migraine (14). However, another study found that serum urate in migraine might be more than control group, which had not shown the results of statistical hypothesis testing (15). Moreover, the reports had never controlled the confounders, which were mainly iron and ferritin, acted as pro-oxidants or anti-oxidants (16). Here, we take the advantage of data from the National Health and Nutrition Examination Survey (NHANES) to clarify the association between serum urate and migraine and explore the dose effect of serum urate in migraine.

## Materials and Methods

### Study Population

Data analyzed in this study were acquired from NHANES (1999–2018), which was designed to assess the nutritional status and health of children and adults in the United States. The program of NHANES was reviewed and approved by the Prevention National Center for Health Statistics Research (NCHS) and the Centers for Disease Control (CDC) Research Ethics Review Board (17), and all participants signed written informed consent (17).

The data release cycles with diseases which were encoded with the International Classification of Diseases, Tenth Revision, Clinical Modification (ICD-10-CM) were enrolled. The participants enrolled without data on serum uric acid (sUA), serum iron, and hemoglobin were excluded.

### Variables

#### Migraine Definition

The identification of migraine cases was based on the self-reported questionnaire (18–20). The procedures were as followed. First, the survey participants were asked about the usage of prescription medications according to the prescription medication questionnaire, which was summarized in the supplementary material. Then, the interviewers used Lexicon Plus^®^ to organize the prescription medicine and used ICD-10-CM to encode the diseases, which were the reasons for the usage of prescription medicine. Finally, we identified the migraine cases on the open information of prescription medication of NHANES, whose ICD-10-CM encode was G43 or G43.P.

#### Exposure Variable

Serum urate was the exposure variable of this cross-section study. Besides, the serum urate was measured by the Beckman Synchron LX20. The covariates included in the study were as follows: age, gender, race, data release cycle, drug usage, blood albumin, blood globulin, blood total protein, hemoglobin, blood creatinine, blood urea nitrogen, ferritin, serum iron, urine albumin, urine creatinine, and urine albumin/creatinine ratio.

### Statistical Methods

Empower software (www.empowerstats.com; X&Y solutions, Inc., Boston MA) was utilized for data analysis. The NHANES sample weights had been applied to all estimates of the study. Continuous variables were expressed as mean ± standard deviation (SD), and the difference test between groups was calculated by a weighted linear regression model. Meanwhile, categorical variables were expressed as percentage, and the difference test of groups was calculated by weighted chi-square test. Logistic regression models were applied to estimate the independent correlation between migraine and serum urate before or after adjustment of confounders. Moreover, when a non-linear relationship between serum urate and the risk of migraine existed, smooth curve fittings were used to examine whether the independent variables were partitioned into intervals (21). When partitioned intervals existed, the inflection point was calculated according to the two-piecewise linear regression model (21, 22). A log-likelihood ratio test, which compared a standard linear regression model to a two-piecewise linear regression model, was used to examine whether a threshold existed (22). In addition, the value of *p* not more than 0.05 was set as a significant level.

## Results

### Description of Study Participants

**Figure 1:**
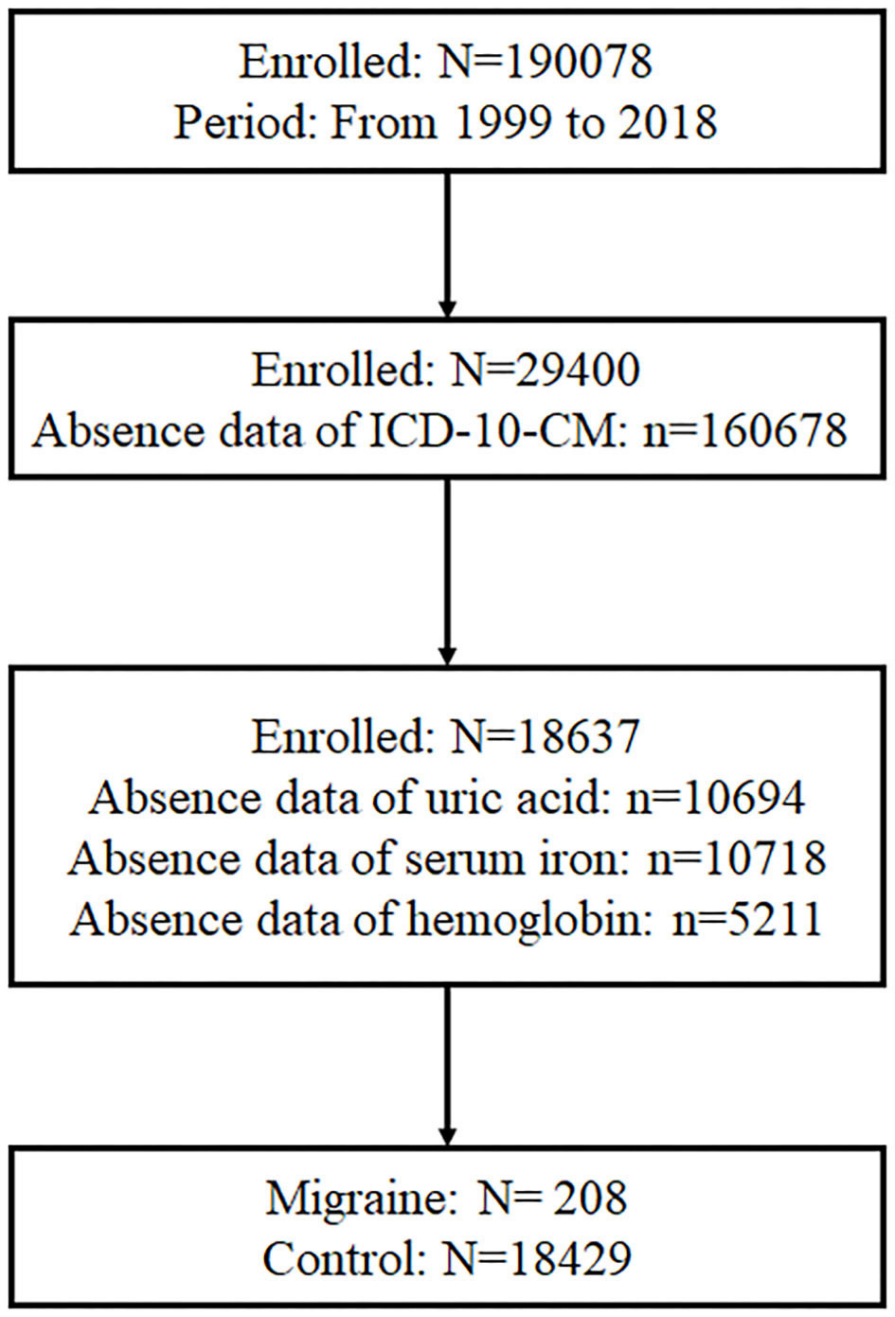
Participants screening procedure. In total, 190,078 participants participated in the NHANES from 1999 to 2018, and 29,400 participants had information about ICD-10-CM. Of the 29,400 participants, 18,637 participants had data of serum urate, serum iron, and hemoglobin. Besides, 208 participants suffered from migraine and had prescription medications for migraine.

As displayed in Figure 1, there were 190,078 participants who participated in the NHANES from 1999 to 2018, and 29,400 participants had information of ICD-10-CM. Of the 29,400 participants, 18,637 participants had data on serum urate, serum iron, and hemoglobin. Meanwhile, 208 participants suffered from migraine and had prescription medications for migraine, and the rest of participants without migraine were set as control.

### Baseline Characteristic of Study Participants

In the study, the mean age of the migraine group and the control group was 46.54 ± 15.01 and 44.15 ± 19.20 years, respectively. Meanwhile, there were 22.73% of men in the migraine group and 48.81% of men in the control group. There were statistically significant differences in the mean age and gender between the two groups. Besides, statistically significant differences were found in the distribution of races (*p* <0.0001) and data release cycles (*p* <0.048) between the two groups. Meanwhile, the migraine group had a higher rate of usage of non-steroidal anti-inflammatory drugs (NSAIDs) (36.14 vs. 5.84%, *p* <0.0001), opioid (6.71 vs. 2.35%, *p* <0.0001), triptans (34.32 vs. 0%, *p* <0.0001), antiepileptic drugs (48.21 vs. 3.27%, *p* <0.0001), antidepressants (35.88 vs. 6.17%, *p* <0.0001), β-blockers (20.42 vs. 7.50%, *p* <0.0001), Ca^2+^ blockers (8.83 vs. 5.41%, *p* = 0.013), and antihistamines (7.62 vs. 0.28%, *p* <0.0001), as compared with the control group. Furthermore, the migraine group had the lower values of blood albumin (4.17 vs. 4.26, *p* <0.0001), blood total protein (6.95 vs. 7.10, *p* <0.0001), hemoglobin (13.73 vs. 14.15, *p* <0.0001), serum iron (77.39 vs. 86.26, *p* <0.0001), and serum urate (4.86 vs. 5.36, *p* <0.0001), as compared with the control group. However, there were no differences in blood globulin, blood creatinine, blood urea nitrogen, ferritin, urine albumin, urine creatinine, or urine albumin/creatinine ratio. The data are shown in Table 1.

**Table 1:** Characteristic of participants enrolled.

	**Control** **(*n* = 18,429)**	**Migraine** **(*n* = 208)**	***P***
Age	44.15 ± 19.20	46.54 ± 15.01	*0.0389*
**Gender**			* <0.0001*
Male	48.81%	22.73%	
Female	51.19%	77.27%	
**Race**			* <0.0001*
Mexican American	9.89%	5.72%	
Other Hispanic	6.49%	2.41%	
Non-Hispanic White	63.06%	78.73%	
Non-Hispanic Black	11.20%	6.56%	
Other Race	9.36%	6.58%	
**Data release cycle**			*0.048*
8	33.41%	36.08%	
9	33.31%	26.34%	
10	33.28%	37.58%	
**Drug usage**			
NSAIDs^*^	5.84%	36.14%	* <0.0001*
Opioid	2.35%	6.71%	* <0.0001*
Triptans	0.00%	34.32%	* <0.0001*
Antiepileptic drugs	3.27%	48.21%	* <0.0001*
Antidepressants	6.17%	35.88%	* <0.0001*
Beta-blockers	7.50%	20.42%	* <0.0001*
Ca^2+^ blockers	5.41%	8.83%	*0.013*
Antihistamines	0.28%	7.62%	* <0.0001*
Blood albumin (g/dL)	4.26 ± 0.36	4.17 ± 0.33	* <0.0001*
Blood globulin(g/dL)	2.84 ± 0.43	2.79 ± 0.44	0.0659
Blood total protein (g/dL)	7.10 ± 0.44	6.95 ± 0.45	* <0.0001*
Hemoglobin(g/dL)	14.15 ± 1.46	13.73 ± 1.35	* <0.0001*
Blood creatinine (mg/dL)	0.87 ± 0.37	0.83 ± 0.21	0.1257
Blood urea nitrogen (mg/dL)	14.01 ± 5.43	13.38 ± 4.66	0.0553
Ferritin (ng/ml)	119.64 ± 97.97	118.77 ± 84.57	0.883
Serum iron (ug/dl)	86.26 ± 36.22	77.39 ± 30.97	* <0.0001*
Urine albumin (ug/ml)	37.05 ± 259.07	23.09 ± 59.56	0.371
Urine creatinine (mg/dL)	124.51 ± 81.42	122.56 ± 82.02	0.6935
Urine albumin/creatinine ratio(mg/g)	35.26 ± 286.76	19.98 ± 64.26	0.3764
Serum urate (mg/dL)	5.36 ± 1.40	4.86 ± 1.45	* <0.0001*

*Continuous variables were shown as mean ± standard deviation (SD), and the value of p was calculated by the weighted linear regression model. Categorical variables were shown as percentage, and the p was calculated by weighted chi-square test.*

* *NSAIDs: non-steroidal anti-inflammatory drugs*.

### The Relationship Between Serum Urate and Migraine

The results of logistic regression models are shown in Table 2. Model 1 was a non-adjusted model, and model 2 was adjusted for age, gender, race, and data release cycle. Meanwhile, model 3 was adjusted for age, gender, race, data release cycle, blood albumin, blood globulin, blood total protein, hemoglobin, blood urea nitrogen, ferritin, serum iron, usage of NSAIDs, usage of opioid, usage of triptans, usage of antiepileptic drugs, usage of antidepressants, usage of β-blockers, usage of Ca^2+^ blockers, and usage of antihistamines. Although we found that lower serum urate was correlated to higher migraine occurrence in model 1 (odds ratio (*OR*) = 0.832, 95% confidence interval (*CI*) = (0.751, 0.922), *p* = 0.00044) and model 3 (*OR* = 0.84, 95% *CI* = (0.72, 0.97), *p* = *0.0184*), the results were not consistent in model 2 (*OR* = 0.96, 95% *CI* = (0.86, 1.08), *p* = 0.5198). Furthermore, in the subgroup analysis, which was stratified by gender, we found that there was no correlation between migraine and serum urate, except for model 3 (*OR* = 0.79, 95% *CI* = (0.67, 0.94), *p* = 0.0092*)* in women. Meanwhile, when stratified by race, we found that a negative correlation between migraine and serum urate existed in non-Hispanic White. However, a positive correlation existed in other races. These results demonstrated that there was no relationship or the relationship was non-linear between migraine and serum urate.

**Table 2:** Association between serum urate and migraine.

	**Model 1**	**Model 2**	**Model 3**
	OR^*^(95%CI^#^) *P*	**OR (95%CI) *P***	**OR (95%CI) *P***
Serum urate (mg/dL)	0.83 (0.75, 0.92) *0.0004*	0.96 (0.86, 1.08) 0.5198	0.84 (0.72, 0.97) *0.0184*
**Stratified by gender**			
Male	0.99 (0.79, 1.24) 0.9442	0.98 (0.78, 1.23) 0.8494	1.02 (0.74, 1.40) 0.8982
Female	0.99 (0.88, 1.12) 0.8518	0.96 (0.85, 1.09) 0.5307	0.79 (0.67, 0.94) *0.0092*
**Stratified by race**			
Mexican American	0.85 (0.63, 1.15) 0.2940	1.05 (0.76, 1.43) 0.7854	0.86 (0.55, 1.34) 0.5030
Other Hispanic	0.71 (0.46, 1.10) 0.1274	0.97 (0.60, 1.55) 0.8830	0.62 (0.31, 1.24) 0.1743
Non-Hispanic White	0.72 (0.62, 0.84) * <0.0001*	0.83 (0.71, 0.97) *0.0216*	0.79 (0.64, 0.97) *0.0279*
Non-Hispanic Black	0.88 (0.69, 1.13) 0.3135	1.00 (0.77, 1.31) 0.9805	0.89 (0.61, 1.29) 0.5242
Other races	1.21 (0.94, 1.56) 0.1313	1.48 (1.13, 1.94) *0.0050*	1.02 (0.60, 1.73) 0.9415

*Model 1 was adjusted for nothing.*

*Model 2 was adjusted for age, gender, race, and data cycle release.*

*Model 3 was adjusted for age, gender, race, data release cycle, blood albumin, blood globulin, blood total protein, hemoglobin, blood urea nitrogen, ferritin, serum iron, usage of NSAIDs^&^, usage of opioid, usage of triptans, usage of antiepileptic drugs, usage of antidepressants, usage of β-blockers, usage of Ca^2+^ blockers, and usage of antihistamines.*

* *OR: odds ratio.*

# *CI: confidence interval. ^&^NSAIDs: non-steroidal anti-inflammatory drugs*.

**Figure 2:**
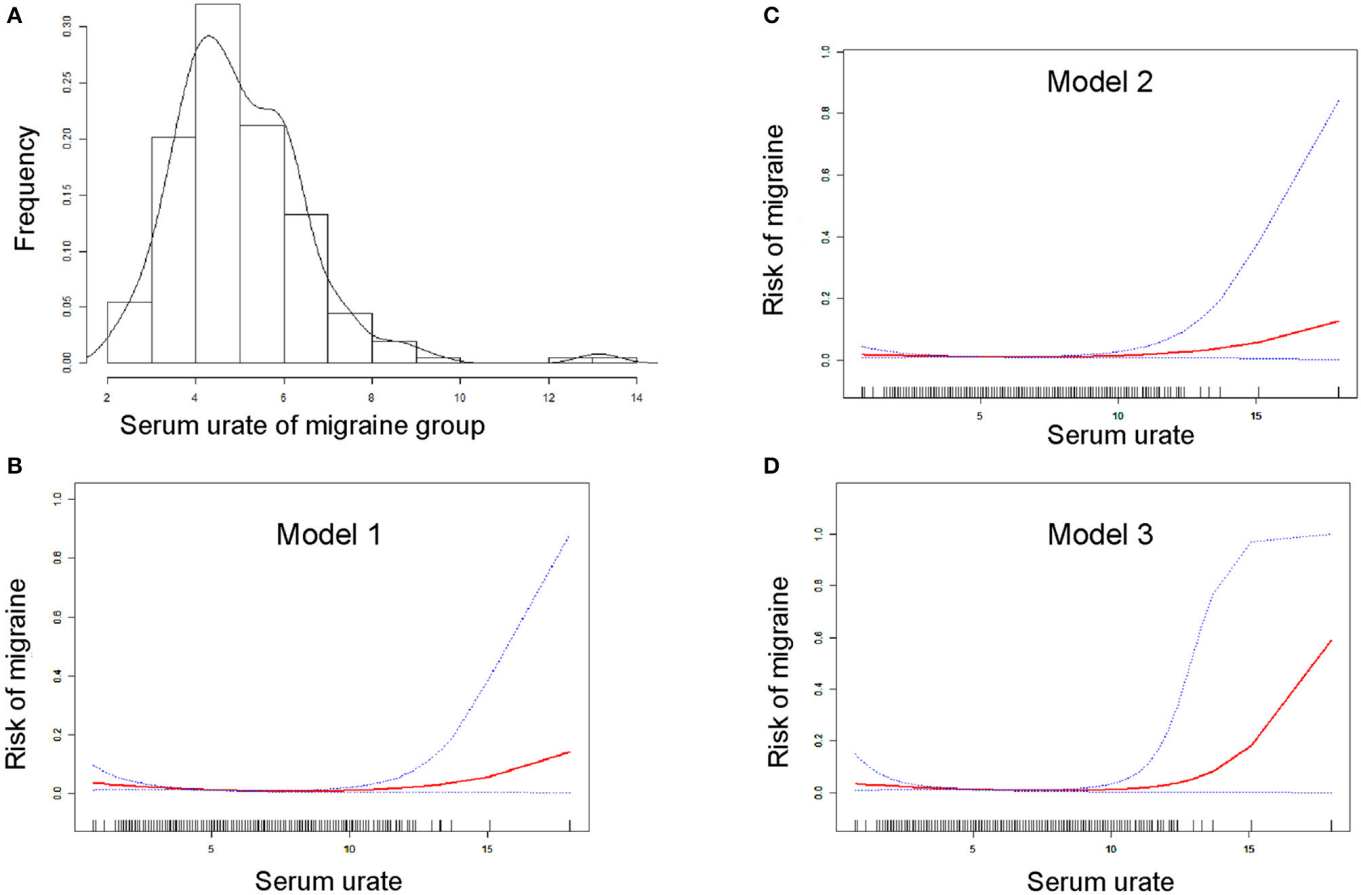
Smooth curve fitting for the relationship between serum urate and the risk of migraine. **(A)** Shows the bell curve of serum urate of migraine group. **(B–D)** Show the smooth curve fitting for the relationship between serum urate and the risk of migraine. The horizontal axis presents serum urate (continuous variable), and the ordinate presents the risk of migraine (0 = non-migraine, 1 = migraine). Red line means smooth curve fitting between serum urate and migraine. Blue bands mean 95% of confidence interval (*CI*) from the fit. Model 1 **(B)** was adjusted for nothing. Model 2 **(C)** was adjusted for age, gender, race, and data release cycle. Model 3 **(D)** was adjusted for age, gender, race, data release cycle, blood albumin, blood globulin, blood total protein, hemoglobin, blood urea nitrogen, ferritin, serum iron, usage of NSAIDs, usage of opioid, usage of triptans, usage of antiepileptic drugs, usage of antidepressants, usage of β-blockers, usage of Ca^2+^ blockers, and usage of antihistamines.

A bell curve found that the data on serum urate in migraineurs fit a normal distribution (Figure 2A). Furthermore, smooth curve fittings were utilized to characterize the non-linear relationship between migraine and serum urate, and found an exponential curve relationship between serum urate and migraine, which is shown in Figure 2. The inflection point was 7.8 mg/dl according to the two-piecewise linear regression model. When serum urate was <7.8 mg/dl, there existed a negative correlation between serum urate and migraine in model 1 (*OR* = 0.77, 95% *CI* = (0.69, 0.86), *p* <0.0001) or model 3 (*OR* = 0.77, 95% *CI* = (0.65, 0.90), *p* = 0.0015). However, there was no correlation between serum urate and migraine in model 2 (*OR* = 0.90, 95% *CI* = (0.80, 1.02), *p* = 0.0872). Furthermore, when serum urate was more than 7.8 mg/dl, higher serum urate was correlated with higher migraine occurrence in model 1(*OR* = 1.54, 95% *CI* = (1.17, 2.04), *p* = 0.0022), model 2 (*OR* = 1.51, 95% *CI*= (1.13, 2.02), *p* = 0.0050), and model 3 (*OR* = 1.77, 95% *CI* = (1.04, 3.02), *p* = 0.0348). In addition, 200 of the 208 (96.1%) migraineurs have a serum urate not more than 7.8 mg/dl, and 17,493 of 18,429 (94.9%) control have a serum urate not more than 7.8 mg/dl. The data are shown in Table 3.

**Table 3:** Threshold effect analysis of serum urate on migraine using the two-piecewise linear regression model.

	**Model 1**	**Model 2**	**Model 3**
	OR^*^(95%CI^#^) *P*	**OR (95%CI) *P***	**OR (95%CI) *P***
Fitting by the standard linear model	0.83 (0.75, 0.92) *0.0004*	0.96 (0.86, 1.08) 0.5198	0.99 (0.88, 1.11) 0.8620
**Fitting by two-piecewise linear model**			
Inflection point	7.8	7.8	7.8
Serum urate <7.8 mg/dL	0.77 (0.69, 0.86) * <0.0001*	0.90 (0.80, 1.02) *0.0872*	0.77 (0.65, 0.90) *0.0015*
Serum urate >7.8 mg/dL	1.54 (1.17, 2.04) *0.0022*	1.51 (1.13, 2.02) *0.0050*	1.77 (1.04, 3.02) *0.0348*
Log likelihood ratio	*0.002*	*0.014*	*0.005*

*Model 1 was adjusted for nothing.*

*Model 2 was adjusted for age, gender, race, and data cycle release.*

*Model 3 was adjusted for age, gender, race, data release cycle, blood albumin, blood globulin, blood total protein, hemoglobin, blood urea nitrogen, ferritin, serum iron, usage of NSAIDs^&^, usage of opioid, usage of triptans, usage of antiepileptic drugs, usage of antidepressants, usage of β-blockers, usage of Ca^2+^ blockers, and usage of antihistamines.*

* *OR: odds ratio.*

# *CI: confidence interval. ^&^NSAIDs: non-steroidal anti-inflammatory drugs*.

## Discussion

According to the nationally representative cross-section study of the United States, there existed no consistent linear relationship between serum urate and migraine according to logistic regression models, without or with stratified by gender or race, except in non-Hispanic White. However, we found an exponential curve relationship between serum urate and migraine, with an infliction point of 7.8 mg/dl. When serum urate was more than 7.8 mg/dl, increased serum urate was correlated with higher migraine occurrence. However, when serum urate was <7.8 mg/dl, there was no consistent relationship between serum urate and migraine without or with confounders adjusted. To the best of our knowledge, our study demonstrates that serum urate is a risk factor for migraine for the first time. Our study provides a target and rationale for serum urate control in migraineurs.

Usually, serum urate levels higher than 6 mg/dl lead to the deposition of monosodium urate (MSU) crystal in tendons, joints, or other unusual tissues at physiological pH (~7.4) (23). Meanwhile, previous studies have found that inflammation plays an important role in the pathophysiology of migraine, and the levels of serum inflammatory cytokines, such as CRP, IL-1β, IL-6, and TNF-α are higher in migraineurs compared with healthy controls (24). Moreover, NSAIDs and corticosteroids are used for shortening a migraine attack (25, 26). Serum urate has been proposed as a neuroprotective agent in stroke, Parkinson's disease, multiple sclerosis, and Alzheimer's disease, in which the high serum urate levels have been linked to the lower severity of neurological injury (13). However, studies in animal or human had failed to prove the neuroprotective effect of serum urate by regulating the serum urate levels (13). A previous study found that migraineurs had the lower levels of serum urate and ferritin, as compared with healthy controls (14). Meanwhile, there existed no statistically significant differences between the different subtypes of migraine, which compared migraine with/without aura or episodic/chronic migraine (14). Another study, which was aimed at assessing the change of serum urate in migraineurs receiving topiramate, found that the serum urate of migraine pretreatment with topiramate and the well-matched control group were 3.61 ± 0.89 and 3.09 ± 1.86, respectively (15). Besides, topiramate would increase the serum urate level in migraine (15). The normal range of serum urate in humans is 2.5–7.0 mg/dl in men and 1.5–6.0 mg/dl in women (13). Hyperuricemia is defined as a serum urate more than 6.8 mg/dl, which mostly individuals would suffer from gout (13). Furthermore, hyperuricemia is a risk factor for hypercholesterolemia, diabetes, hypertension, cardiovascular and cerebrovascular events, and so on (13). In the present study, the migraine group had lower serum urate before adjusting for confounders. However, the linear relationship was not consistent after adjusting for confounders. Furthermore, an exponential curve relationship, with an infliction point of 7.8 mg/dl, was found in serum urate and the risk of migraine. Our findings demonstrated that serum urate, when more than 7.8 mg/dl, might be a risk factor for migraine. Furthermore, we also suggest that there is a threshold effect on the neuroprotective effect and that the infliction point might be 7.8 mg/dl. Besides, the underlining mechanism might be that MSU crystal formed might trigger inflammation *via* IL-1b, TNF-a, IL-6, IL-8, and oxidative stress (27–31).

Currently, there are a lot of risk factors for migraine identified by researchers. It had been found that 38 genetic loci, which were enriched in vascular biology, were associated with migraine (4, 32). Meanwhile, another study had found that the genetically mediated hypercalcemia might increase the risk of migraine (32, 33). In addition, migraine with aura was associated with the increasing risk of other comorbidities, such as perioperative stroke, patent foramen ovale, and restless legs syndrome (32). Risk factors associated with the progression from episodic migraine to chronic migraine were summarized in a previous review (34, 35). Besides the fair and non-modifiable risk factors included female gender, low family socioeconomic status, and major life events (34, 35). Furthermore, the moderate and modifiable risk factors were obesity, persistent-frequent nausea associated with migraine, asthma, non-cephalic pain, snoring, and the efficacy of abortive migraine treatments (34, 35). Moreover, strong and modifiable risk factors were the frequency of headache day, depression, and acute medication use/overuse (34, 35). In our analysis, we found that the serum urate levels of more than 7.8 mg/dl might be a risk factor for migraine, which is a modifiable risk factor.

The limitations of this study were as follows: first, the present study could not distinguish the acute attack of migraine, the frequency and intensity of migraine attack, and migraine with/without aura, because of the missing information of NHANES. Second, the present study was a cross-section study, whose follow-up data were absent. Besides, the conclusions needed to be proven according to a prospective longitudinal study in the future, which should control the confounders, such as age, race, gender, intensity and frequency of migraine attack, and drug usage. Finally, the medication usage was analyzed in our analysis, but the dosages of drugs were unknown. Further studies could be conducted to investigate the alteration of serum urate levels in migraineurs with different drugs in real world studies.

In conclusion, there existed an exponential curve relationship between serum urate and migraine, with an infliction point of 7.8 mg/dl. When the serum urate was more than 7.8 mg/dl, increased serum urate was correlated with higher migraine occurrence.

## Data Availability Statement

Publicly available datasets were analyzed in this study. This data can be found here: Centers for Disease Control and Prevention (CDC) National Health and Nutrition Examination Survey (NHANES), https://wwwn.cdc.gov/nchs/nhanes/Default.aspx, NHANES 1999-2018.

## Ethics Statement

The studies involving human participants were reviewed and approved by the Prevention National Center for Health Statistics Research (NCHS) and Centers for Disease Control (CDC) Research Ethics Review Board. Written informed consent to participate in this study was provided by the participants' legal guardian/next of kin.

## Author Contributions

YX, HX, and YW proposed the idea. PH and YL acquired the data. PH analyzed the data. YX wrote the first draft. HX, YW, and PH revised the draft. All authors have approved the final article.

## Conflict of Interest

The authors declare that the research was conducted in the absence of any commercial or financial relationships that could be construed as a potential conflict of interest.

## Publisher's Note

All claims expressed in this article are solely those of the authors and do not necessarily represent those of their affiliated organizations, or those of the publisher, the editors and the reviewers. Any product that may be evaluated in this article, or claim that may be made by its manufacturer, is not guaranteed or endorsed by the publisher.
